# Multi-state Modeling of Biomolecules

**DOI:** 10.1371/journal.pcbi.1003844

**Published:** 2014-09-25

**Authors:** Melanie I. Stefan, Thomas M. Bartol, Terrence J. Sejnowski, Mary B. Kennedy

**Affiliations:** 1 Division of Biology and Biological Engineering, California Institute of Technology, Pasadena, California, United States of America; 2 Salk Institute for Biological Studies, La Jolla, California, United States of America; 3 Division of Biology and Biological Engineering, California Institute of Technology, Pasadena, California, United States of America; University of Toronto, Canada

## Abstract

Multi-state modeling of biomolecules refers to a series of techniques used to represent and compute the behavior of biological molecules or complexes that can adopt a large number of possible functional states. Biological signaling systems often rely on complexes of biological macromolecules that can undergo several functionally significant modifications that are mutually compatible. Thus, they can exist in a very large number of functionally different states. Modeling such multi-state systems poses two problems: the problem of how to describe and specify a multi-state system (the “specification problem”) and the problem of how to use a computer to simulate the progress of the system over time (the “computation problem”). To address the specification problem, modelers have in recent years moved away from explicit specification of all possible states and towards rule-based formalisms that allow for implicit model specification, including the κ-calculus [1], BioNetGen [2]–[5], the Allosteric Network Compiler [6], and others [7], [8]. To tackle the computation problem, they have turned to particle-based methods that have in many cases proved more computationally efficient than population-based methods based on ordinary differential equations, partial differential equations, or the Gillespie stochastic simulation algorithm [9], [10]. Given current computing technology, particle-based methods are sometimes the only possible option. Particle-based simulators fall into two further categories: nonspatial simulators, such as StochSim [11], DYNSTOC [12], RuleMonkey [9], [13], and the Network-Free Stochastic Simulator (NFSim) [14], and spatial simulators, including Meredys [15], SRSim [16], [17], and MCell [18]–[20]. Modelers can thus choose from a variety of tools, the best choice depending on the particular problem. Development of faster and more powerful methods is ongoing, promising the ability to simulate ever more complex signaling processes in the future.

This is a “Topic Page” article for *PLOS Computational Biology*.

## Introduction

### Multi-state biomolecules in signal transduction

In living cells, signals are processed by networks of proteins that can act as complex computational devices [21]. These networks rely on the ability of single proteins to exist in a variety of functionally different states achieved through multiple mechanisms, including post-translational modifications, ligand binding, conformational change, or formation of new complexes [21]–[24]. Similarly, nucleic acids can undergo a variety of transformations, including protein binding, binding of other nucleic acids, conformational change, and DNA methylation.

In addition, several types of modifications can coexist, exerting a combined influence on a biological macromolecule at any given time. Thus, a biomolecule or complex of biomolecules can often adopt a very large number of functionally distinct states. The number of states scales exponentially with the number of possible modifications, a phenomenon known as “combinatorial explosion” [24]. This is of concern for computational biologists who model or simulate such biomolecules, because it raises questions about how such large numbers of states can be represented and simulated.

### Examples of combinatorial explosion

Biological signaling networks incorporate a wide array of reversible interactions, post-translational modifications, and conformational changes. Furthermore, it is common for a protein to be composed of several—identical or nonidentical—subunits and for several proteins and/or nucleic acid species to assemble into larger complexes. A molecular species with several of those features can therefore exist in a large number of possible states.

For instance, it has been estimated that the yeast scaffold protein Ste5 can be a part of 25,666 unique protein complexes [22]. In E. coli, chemotaxis receptors of four different kinds interact in groups of three, and each individual receptor can exist in at least two possible conformations and has up to eight methylation sites [23], resulting in more than 10^9^ potential states. The Ca2+/calmodulin-dependent protein kinase II (CaMKII) is a dodecamer of twelve catalytic subunits [25], arranged in two hexameric rings [26]. Each subunit can exist in at least two distinct conformations, and each subunit features various phosphorylation and ligand binding sites. A recent model [27] incorporated conformational states, two phosphorylation sites, and two modes of binding calcium/calmodulin, for a total of around 10^9^ possible states per hexameric ring. A model of coupling of the EGF receptor to a mitogen-activated protein (MAP) kinase cascade presented by Danos and colleagues [28] accounts for ∼10^23^ distinct molecular species, yet the authors note several points at which the model could be further extended. A more recent model of ErbB receptor signaling even accounts for more than one googol (10^100^) distinct molecular species [29]. The problem of combinatorial explosion is also relevant to synthetic biology, with a recent model of a relatively simple synthetic eukaryotic gene circuit featuring 187 species and 1,165 reactions [30].

Of course, not all of the possible states of a multi-state molecule or complex will necessarily be populated. Indeed, in systems in which the number of possible states is far greater than that of molecules in the compartment (e.g., the cell), they cannot be. In some cases, empirical information can be used to rule out certain states if, for instance, some combinations of features are incompatible. In the absence of such information, however, all possible states need to be considered a priori. In such cases, computational modeling can be used to uncover to what extent the different states are populated.

It is worth noting that the existence (or potential existence) of such large numbers of molecular species is a combinatorial phenomenon: it arises from a relatively small set of features or modifications (such as post-translational modification or complex formation) that combine to dictate the state of the entire molecule or complex in the same way that the existence of just a few choices in a coffee shop (small, medium, or large; with or without milk; decaf or not; extra shot of espresso) quickly leads to a large number of possible beverages (24 in this case; each additional binary choice will double that number). Although it is difficult for us to grasp the total number of possible combinations, it is usually not conceptually difficult to understand the (much smaller) set of features or modifications and the effect each of them has on the function of the biomolecule. The rate at which a molecule undergoes a particular reaction will usually depend mainly on a single feature or a small subset of features. It is the presence or absence of those features that dictates the reaction rate. The reaction rate is the same for two molecules that differ only in features that do not affect this reaction. Thus, the number of parameters will be much smaller than the number of reactions. (In the coffee shop example, adding an extra shot of espresso will cost 40 cents, no matter what size the beverage is and whether or not it has milk in it). It is such “local rules” that are usually discovered in laboratory experiments. Thus, a multi-state model can be conceptualized in terms of combinations of modular features and local rules. This means that even a model that can account for a vast number of molecular species and reactions is not necessarily conceptually complex.

### Specification versus computation

**Figure 1:**
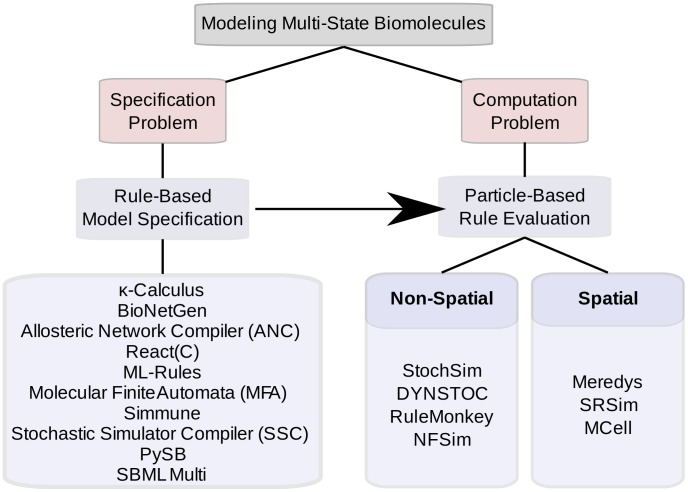
An overview of tools discussed here that are used for the rule-based specification and particle-based evaluation (spatial or nonpatial) of multi-state biomolecules.

The combinatorial complexity of signaling systems involving multi-state proteins poses two kinds of problems. The first problem is concerned with how such a system can be specified, i.e., how a modeler can specify all complexes, all changes those complexes undergo, and all parameters and conditions governing those changes in a robust and efficient way. This problem is called the “specification problem.” The second problem concerns computation. It asks questions about whether a combinatorially complex model, once specified, is computationally tractable given the large number of states and the even larger number of possible transitions between states, whether it can be stored electronically, and whether it can be evaluated in a reasonable amount of computing time. This problem is called the “computation problem.” Among the approaches that have been proposed to tackle combinatorial complexity in multi-state modeling, some are mainly concerned with addressing the specification problem, and some are focused on finding effective methods of computation. Some tools address both specification and computation. The sections below discuss rule-based approaches to the specification problem and particle-based approaches to solving the computation problem. A list of the tools discussed here is presented in Figure 1. A comprehensive overview and discussion of various tools available for multi-state modeling can be found in Chylek et al. [31].

## The Specification Problem

### Explicit specification

The most naïve way of specifying a biomolecule in a biological model is to specify each of its states explicitly and use each of them as a molecular species in a simulation framework that allows transitions from state to state. For instance, if a protein can be ligand or not, exist in two conformational states (e.g., open or closed), and be located in two possible subcellular areas (e.g., cytosolic or membrane), then the eight possible resulting states can be explicitly enumerated as follows:

- bound, open, cytosol
- bound, open, membrane
- bound, closed, cytosol
- bound, closed, membrane
- unbound, open, cytosol
- unbound, open, membrane
- unbound, closed, cytosol
- unbound, closed, membrane

Enumerating all possible states is a lengthy and potentially error-prone process. For macromolecular complexes that can adopt multiple states, enumerating each state quickly becomes tedious, if not impossible. Moreover, the addition of a single additional modification or feature to the model of the complex under investigation will double the number of possible states (if the modification is binary), and it will more than double the number of transitions that need to be specified.

### Rule-based model specification

It is clear that an explicit description, which lists all possible molecular species (including all their possible states), all possible reactions or transitions these species can undergo, and all parameters governing these reactions, very quickly becomes unwieldy as the complexity of the biological system increases. Modelers have therefore looked for implicit, rather than explicit, ways of specifying a biological signaling system. An implicit description is one that groups reactions and parameters that apply to many types of molecular species into one reaction template. It might also add a set of conditions that govern reaction parameters, e.g., the likelihood or rate at which a reaction occurs or whether it occurs at all. Only properties of the molecule or complex that matter to a given reaction (either affecting the reaction or being affected by it) are explicitly mentioned, and all other properties are ignored in the specification of the reaction.

For instance, the rate of ligand dissociation from a protein might depend on the conformational state of the protein but not on its subcellular localization. An implicit description would therefore list two dissociation processes (with different rates, depending on conformational state) but would ignore attributes referring to subcellular localization, because they do not affect the rate of ligand dissociation nor are they affected by it. This specification rule has been summarized as “don't care, don't write” [28].

Since it is not written in terms of reactions but in terms of more general “reaction rules” encompassing sets of reactions, this kind of specification is often called “rule-based” [4]. This description of the system in terms of modular rules relies on the assumption that only a subset of features or attributes are relevant for a particular reaction rule. Where this assumption holds, a set of reactions can be coarse-grained into one reaction rule. This coarse-graining preserves the important properties of the underlying reactions. For instance, if the reactions are based on chemical kinetics, so are the rules derived from them.

Many rule-based specification methods exist. In general, the specification of a model is a separate task from the execution of the simulation. Therefore, among the existing rule-based model specification systems [4], some concentrate on model specification only, allowing the user to then export the specified model into a dedicated simulation engine. However, many solutions to the specification problem also contain a method of interpreting the specified model [3]. This is done by providing a method to simulate the model or a method to convert it into a form that can be used for simulations in other programs.

An early rule-based specification method is the Kappa (κ)-calculus [1], a process algebra that can be used to encode macromolecules with internal states and binding sites and to specify rules by which they interact. A review of κ is provided by Danos et al. [28]. The κ-calculus is merely concerned with providing a language to encode multi-state models, not with interpreting the models themselves. A simulator compatible with Kappa is KaSim [32], [33].

BioNetGen is a software suite that provides both specification and simulation capacities [2]–[5]. Rule-based models can be written down using a specified syntax, the BioNetGen language (BNGL) [4]. The underlying concept is to represent biochemical systems as graphs, in which molecules are represented as nodes (or collections of nodes) and chemical bonds as edges. A reaction rule then corresponds to a graph rewriting rule [3]. BNGL provides a syntax for specifying these graphs and the associated rules as structured strings [4]. BioNetGen can then use these rules to generate ordinary differential equations (ODEs) to describe each biochemical reaction. Alternatively, it can generate a list of all possible species and reactions in the Systems Biology Markup Language (SBML) [34], [35], which can then be exported to simulation software packages that can read SBML. One can also make use of BioNetGen's own ODE-based simulation software and its capability to generate reactions on the fly during a stochastic simulation [5]. In addition, a model specified in BNGL can be read by other simulation software, such as DYNSTOC [12], RuleMonkey [13], and NFSim [14].

Another tool that generates full reaction networks from a set of rules is the Allosteric Network Compiler (ANC) [6]. Conceptually, ANC sees molecules as allosteric devices with a Monod-Wyman-Changeux (MWC)-type regulation mechanism [36], whose interactions are governed by their internal state, as well as by external modifications. A very useful feature of ANC is that it automatically computes dependent parameters, thereby imposing thermodynamic correctness [37].

An extension of the κ-calculus is provided by React(C) [38]. The authors of React(C) show that it can express the stochastic π calculus [39]. They also provide a stochastic simulation algorithm based on the Gillespie stochastic algorithm [40] for models specified in React(C) [38].

ML-Rules [41] is similar to React(C) but provides the added possibility of nesting: a component species of the model, with all its attributes, can be part of a higher-order component species. This enables ML-Rules to capture multi-level models that can bridge the gap between, for instance, a series of biochemical processes and the macroscopic behavior of a whole cell or group of cells. For instance, Maus et al. have provided a proof-of-concept model of cell division in fission yeast that includes cyclin/cdc2 binding and activation, pheromone secretion and diffusion, cell division, and movement of cells [41]. Models specified in ML-Rules can be simulated using the Java Framework for Modeling and Simulation (JAMES) II [42]. A similar nested language to represent multi-level biological systems has been proposed by Oury and Plotkin [43].

Yang et al. [8] have proposed a specification formalism based on finite automata. Models specified in their Molecular Finite Automata (MFA) framework can then be used to generate and simulate a system of ODEs or for stochastic simulation using a kinetic Monte Carlo algorithm.

Some rule-based specification systems and their associated network generation and simulation tools have been designed to accommodate spatial heterogeneity in order to allow for the realistic simulation of interactions within biological compartments. For instance, the Simmune project [44], [45] includes a spatial component: users can specify their multi-state biomolecules and interactions within membranes or compartments of arbitrary shape. The reaction volume is then divided into interfacing voxels, and a separate reaction network is generated for each of these subvolumes.

The Stochastic Simulator Compiler (SSC) [46] allows for rule-based, modular specification of interacting biomolecules in regions of arbitrarily complex geometries. Again, the system is represented using graphs, with chemical interactions or diffusion events formalized as graph-rewriting rules [46]. The compiler then generates the entire reaction network before launching a stochastic reaction-diffusion algorithm.

A different approach is taken by PySB [47], in which model specification is embedded in the programming language Python. A model (or part of a model) is represented as a Python program. This allows users to store higher-order biochemical processes such as catalysis or polymerization as macros and reuse them as needed. The models can be simulated and analyzed using Python libraries, but PySB models can also be exported into BNGL [4], Kappa [1], and SBML [34].

Models involving multi-state and multi-component species can also be specified in level 3 of the SBML [34] using the multi package. A draft specification is available [48], and software support is under development.

Thus, by only considering states and features important for a particular reaction, rule-based model specification eliminates the need to explicitly enumerate every possible molecular state that can undergo a similar reaction and thereby allows for efficient specification.

## The Computation Problem

When running simulations on a biological model, any simulation software evaluates a set of rules, starting from a specified set of initial conditions and usually iterating through a series of time steps until a specified end time. One way to classify simulation algorithms is by looking at the level of analysis at which the rules are applied: they can be population-based, single-particle-based, or hybrid.

### Population-based rule evaluation

In population-based rule evaluation, rules are applied to populations. All molecules of the same species in the same state are pooled together. Application of a specific rule reduces or increases the size of one of the pools, possibly at the expense of another.

Some of the best-known classes of simulation approaches in computational biology belong to the population-based family, including those based on the numerical integration of ordinary and partial differential equations and the Gillespie stochastic simulation algorithm.

Differential equations describe changes in molecular concentrations over time in a deterministic manner. Simulations based on differential equations usually do not attempt to solve those equations analytically but employ a suitable numerical solver.

The stochastic Gillespie algorithm changes the composition of pools of molecules through a progression of random reaction events, the probability of which is computed from reaction rates and from the numbers of molecules, in accordance with the stochastic master equation [40].

In population-based approaches, one can think of the system being modeled as being in a given state at any given time point, where a state is defined according to the nature and size of the populated pools of molecules. This means that the space of all possible states can become very large. With some simulation methods implementing numerical integration of ordinary and partial differential equations or the Gillespie stochastic algorithm, all possible pools of molecules and the reactions they undergo are defined at the start of the simulation, even if they are empty. Such “generate-first” methods [4] scale poorly with increasing numbers of molecular states [49]. For instance, it has recently been estimated that even for a simple model of CaMKII with just six states per subunits and ten subunits, it would take 290 years to generate the entire reaction network on a 2.54 GHz Intel Xeon processor [50]. In addition, the model generation step in generate-first methods does not necessarily terminate, for instance, when the model includes assembly of proteins into complexes of arbitrarily large size, such as actin filaments. In these cases, a termination condition needs to be specified by the user [3], [5].

Even if a large reaction system can be successfully generated, its simulation using population-based rule evaluation can run into computational limits. In a recent study, a powerful computer was shown to be unable to simulate a protein with more than eight phosphorylation sites (2^8^ = 256 phosphorylation states) using ordinary differential equations [14].

Methods have been proposed to reduce the size of the state space. One is to consider only the states adjacent to the present state (i.e., the states that can be reached within the next iteration) at each time point. This eliminates the need for enumerating all possible states at the beginning. Instead, reactions are generated “on the fly” [4] at each iteration. These methods are available both for stochastic and deterministic algorithms. These methods still rely on the definition of an (albeit reduced) reaction network—in contrast to the “network-free” methods discussed below.

Even with “on-the-fly” network generation, networks generated for population-based rule evaluation can become quite large and thus difficult—if not impossible—to handle computationally. An alternative approach is provided by particle-based rule evaluation.

### Particle-based rule evaluation

In particle-based (sometimes called “agent-based”) simulations, proteins, nucleic acids, macromolecular complexes, or small molecules are represented as individual software objects, and their progress is tracked through the course of the entire simulation [51]. Because particle-based rule evaluation keeps track of individual particles rather than populations, it comes at a higher computational cost when modeling systems with a high total number of particles but a small number of kinds (or pools) of particles [51]. In cases of combinatorial complexity, however, the modeling of individual particles is an advantage because, at any given point in the simulation, only existing molecules, their states, and the reactions they can undergo need to be considered. Particle-based rule evaluation does not require the generation of complete or partial reaction networks at the start of the simulation or at any other point in the simulation and is therefore called “network-free.”

**Figure 2:**
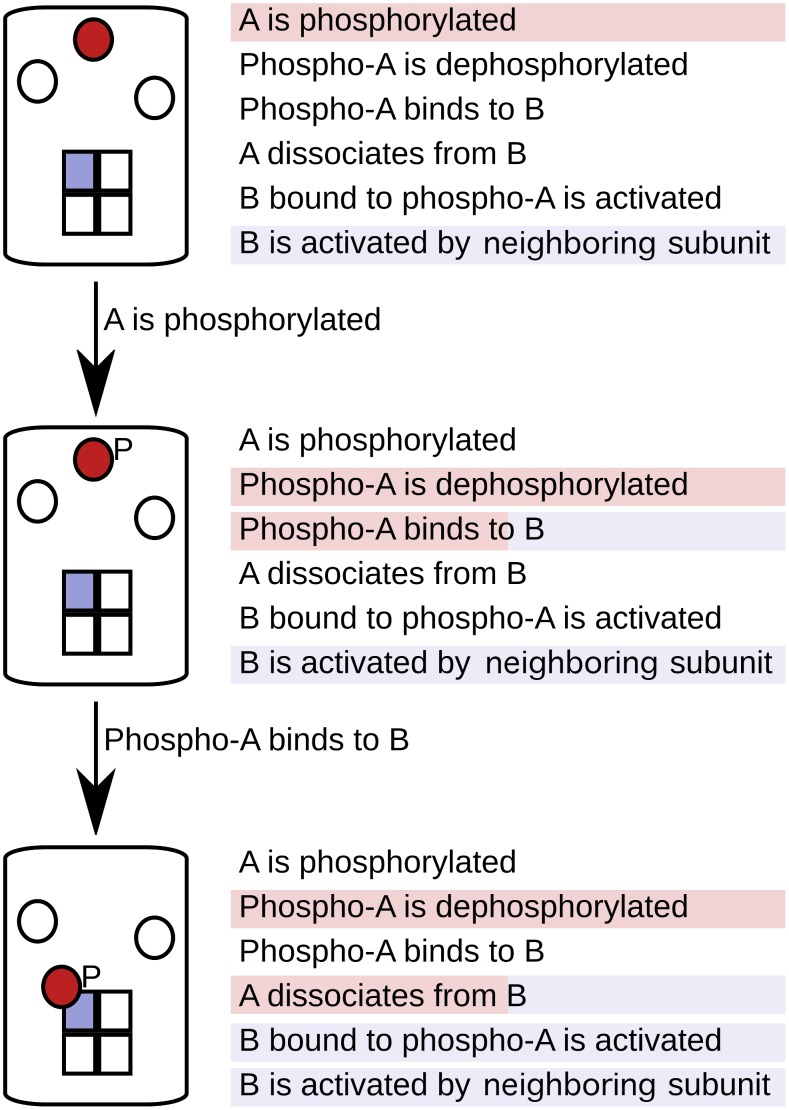
Principles of particle-based modeling. In particle-based modeling, each particle is tracked individually through the simulation. At any point, a particle only “sees” the rules that apply to it. This figure follows two molecular particles (one of type A in red, one of type B in blue) through three steps in a hypothetical simulation following a simple set of rules (given on the right). At each step, the rules that potentially apply to the particle under consideration are highlighted in that particle's colour.

This method reduces the complexity of the model at the simulation stage and thereby saves time and computational power [9]. A detailed discussion of the computational cost of population-based versus particle-based methods is provided in a recent study by Hogg et al. [10]. The simulation follows each particle, and at each simulation step, a particle only “sees” the reactions (or rules) that apply to it. This depends on the state of the particle and, in some implementations, on the states of its neighbors in a holoenzyme or complex. As the simulation proceeds, the states of particles are updated according to the rules that are fired. Figure 2 illustrates the process of particle-based modeling using a simple system with three molecules of type A and one molecular tetramer of type B. This system goes through three simulation steps following a simple set of rules.

Some particle-based simulation packages use an ad hoc formalism for specification of reactants, parameters, and rules. Others can read files in a recognized rule-based specification format such as BNGL [4].

### Nonspatial particle-based methods

StochSim [11], [52] is a particle-based stochastic simulator used mainly to model chemical reactions and other molecular transitions. The algorithm used in StochSim is different from the more widely known Gillespie stochastic algorithm [40] in that it operates on individual entities, not entity pools, making it particle-based rather than population-based.

In StochSim, each molecular species can be equipped with a number of binary state flags representing a particular modification. Reactions can be made dependent on a set of state flags set to particular values. In addition, the outcome of a reaction can include a state flag being changed. Moreover, entities can be arranged in geometric arrays (for instance, for holoenzymes consisting of several subunits), and reactions can be “neighbor-sensitive”, i.e., the probability of a reaction for a given entity is affected by the value of a state flag on a neighboring entity. These properties make StochSim ideally suited to modeling multi-state molecules arranged in holoenzymes or complexes of specified size. Indeed, StochSim has been used to model clusters of bacterial chemotactic receptors [53] and CaMKII holoenzymes [27].

An extension to StochSim has been presented by Colvin et al. [12]. Their particle-based simulator DYNSTOC uses a StochSim-like algorithm to simulate models specified in BNGL [4], which improves the handling of molecules within macromolecular complexes [12].

Another particle-based stochastic simulator that can read BNGL input files is RuleMonkey [13]. Its simulation algorithm [9] differs from the algorithms underlying both StochSim and DYNSTOC in that the simulation time step is variable.

NFSim differs from those described above by allowing for the definition of reaction rates as arbitrary mathematical or conditional expressions and thereby facilitates selective coarse-graining of models [14]. RuleMonkey and NFSim implement distinct but related simulation algorithms. A detailed review and comparison of both tools is given by Yang and Hlavacek [54].

It is easy to imagine a biological system in which some components are complex multi-state molecules, whereas others have few possible states (or even just one) and exist in large numbers. A hybrid approach has been proposed to model such systems: within the hybrid particle/population (HPP) framework, the user can specify a rule-based model but can designate some species to be treated as populations (rather than particles) in the subsequent simulation [10]. This method combines the computational advantages of particle-based modeling for multi-state systems with relatively low molecule numbers and of population-based modeling for systems with high molecule numbers and a small number of possible states. Specification of HPP models is supported by BioNetGen [4], and simulations can be performed with NFSim [14].

### Spatial particle-based methods

Spatial particle-based methods differ from the methods described above by their explicit representation of space.

One example of a particle-based simulator that allows for a representation of cellular compartments is SRSim [16], [17]. SRSim is integrated in the Large-scale Atomic/Molecular Massively Parallel Simulator (LAMMPS) [55], [56] and allows the user to specify the model in BNGL [4]. SRSim allows users to specify the geometry of the particles in the simulation, as well as interaction sites. It is therefore especially good at simulating the assembly and structure of complex biomolecular complexes, as evidenced by a recent model of the inner kinetochore [57].

**Figure 3:**
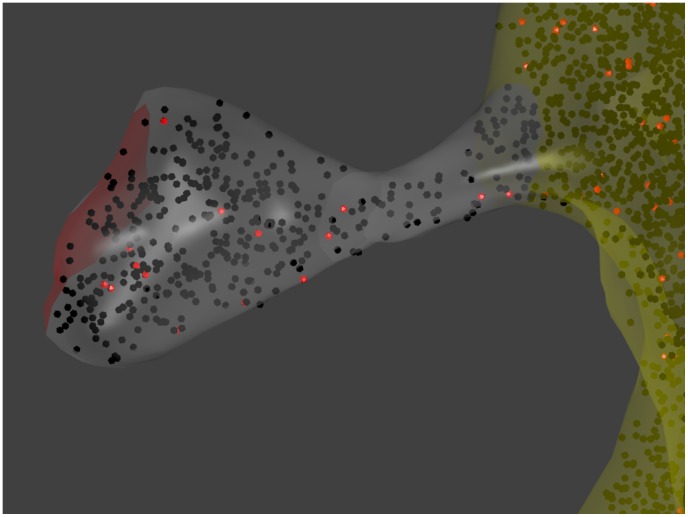
Screenshot from an MCell simulation of calcium signaling within the spine. Although other types of calcium-regulated molecules were included in the simulations, only CaMKII molecules are visualized. They are shown in red when bound to calmodulin and in black when unbound. The simulation compartment is a reconstruction of a dendritic spine as presented by Kinney et al. [59]. The area of the postsynaptic density is shown in red, the spine head and neck in gray, and the parent dendrite in yellow. The figure was generated by visualizing the simulation results in Blender.

MCell [18]–[20], [58] allows individual molecules to be traced in arbitrarily complex geometric environments that are defined by the user. This allows for simulations of biomolecules in realistic reconstructions of living cells, including cells with complex geometries like those of neurons. As an illustration, Figure 3 shows a screenshot from a simulation of calcium proteins. The reaction compartment is a reconstruction of a dendritic spine [59]. Visualizations are supported by a specialized plug-in (“CellBlender”) for the open-source program Blender [60].

MCell uses an ad hoc formalism within MCell itself to specify a multi-state model: in MCell, it is possible to assign “slots” to any molecular species. Each slot stands for a particular modification, and any number of slots can be assigned to a molecule. Each slot can be occupied by a particular state. The states are not necessarily binary. For instance, a slot describing binding of a particular ligand to a protein of interest could take the states “unbound,” “partially bound,” and “fully bound.”

The slot-and-state syntax in MCell can also be used to model multimeric proteins or macromolecular complexes. When used in this way, a slot is a placeholder for a subunit or a molecular component of a complex, and the state of the slot will indicate whether a specific protein component is absent or present in the complex. A way to think about this is that MCell macromolecules can have several dimensions: a “state dimension” and one or more “spatial dimensions.” The “state dimension” is used to describe the multiple possible states making up a multi-state protein, while the spatial dimension(s) describes topological relationships between neighboring subunits or members of a macromolecular complex. One drawback of this method for representing protein complexes, compared to other spatial modeling tools such as Meredys [15], is that MCell does not allow for the diffusion of complexes and hence of multi-state molecules. This can in some cases be circumvented by adjusting the diffusion constants of ligands that interact with the complex by using checkpointing functions or by combining simulations at different levels.

## Examples of Multi-state Models in Biology

A (by no means exhaustive) selection of models of biological systems involving multi-state molecules and using some of the tools discussed here is given in Table 1.

**Table 1:** Examples of multi-state models of biological systems.

Biological system	Specification	Computation	Reference
Bacterial chemotaxis signaling pathway	StochSim	StochSim	[61]
CaMKII regulation	StochSim	StochSim	[27]
ERBB receptor signaling	BioNetGen	NFSim	[29]
Eukaryotic synthetic gene circuits	BioNetGen, PROMOT [62]	COPASI [63]	[30]
RNA signaling	Kappa	KaSim	[64]
Cooperativity of allosteric proteins	ANC	Matlab	[6]
Chemosensingin Dictyostelium	Simmune	Simmune	[44]
T cell receptor activation	SSC	SSC	[65]
Human mitotic kinetochore	BioNetGen	SRSim	[66]
Cell cycle of fission yeast	ML-Rules	JAMES II [42]	[41]

A version of this table with hyperlinks is attached to this manuscript as Table S1. Abbreviations: COPASI, COmplex PAthway Simulator; PROMOT, Process Modeling Tool.

## Supporting Information

Table S1 Table 1 with hyperlinks.
(DOCX)

Text S1 Version history of the text file.
(XML)

Text S2 Peer reviews and response to reviews. Human-readable versions of the reviews and authors responses are available as comments on this article.
(XML)
