# Supplementary material for: Multi-state Modeling of Biomolecules
Source: PLoS Comput Biol. 2014 Sep 25;10(9):e1003844. doi: 10.1371/journal.pcbi.1003844 (PMC4201162; doi:10.1371/journal.pcbi.1003844)
Supplement: Table S1 — Table 1 with hyperlinks. (DOCX) [file pcbi.1003844.s001.docx]

**Table S1: Examples of multi-state models of biological systems**

| Biological system | Specification | Computation | Reference |
| --- | --- | --- | --- |
| Bacterial chemotaxis signaling pathway | StochSim | StochSim | [61] |
| CaMKII regulation | StochSim | StochSim | [27] |
| [ERBB](http://en.wikipedia.org/w/index.php?title=ErbB) receptor signaling | BioNetGen | NFSim | [29] |
| Eukaryotic synthetic gene circuits | BioNetGen, PROMOT [62] | COPASI [63] | [30] |
| RNA signaling | Kappa | KaSim | [64] |
| [Cooperativity](http://en.wikipedia.org/w/index.php?title=cooperative_binding) of allosteric proteins | Allosteric Network Compiler (ANC) | Matlab | [6] |
| [Chemosensing](http://en.wikipedia.org/w/index.php?title=chemoreceptor) in [Dictyostelium](http://en.wikipedia.org/w/index.php?title=Dictyostelium_discoideum) | Simmune | Simmune | [44] |
| [T-cell receptor](http://en.wikipedia.org/w/index.php?title=T-cell_receptor) activation | SSC | SSC | [65] |
| Human mitotic kinetochore | BioNetGen | SRSim | [66] |
| Cell cycle of fission yeast | ML-Rules | JAMES II [42] | [41] |
